# Post-surgical immune suppression: another target to improve postoperative outcomes

**DOI:** 10.1007/s00540-019-02651-3

**Published:** 2019-05-06

**Authors:** Kanji Uchida

**Affiliations:** Department of Anesthesiology, Graduate School of Medicine, The University of Tokyo, 7-3-1, Hongo, Bunkyo-ku, Tokyo, 113-0033 Japan

**Keywords:** Postoperative immune suppression, Sepsis, Immune enhancing therapy

Patients undergoing surgery suffer from “scheduled insult”. In general, surgical insult causes local inflammation via migrating inflammatory cells. Local inflammation is accompanied by systemic inflammatory responses characterized by fever and elevated levels of systemic inflammatory cytokines. In most cases, inflammation eventually subsides, which leads to wound healing. However, for those with severe inflammation it sometimes progresses to sepsis and multiple organ dysfunction syndrome (MODS).

The mortality of sepsis and MODS, which includes acute respiratory distress syndrome (ARDS) and acute kidney injury (AKI), is high and is still regarded as a troublesome condition in critical care settings [1, 2].

Highly-invasive surgeries such as esophagectomy, pancreaticoduodenectomy, vascular surgery, cardiac surgery often associate with infectious complications, which resembles the clinical course of trauma, severe infection and sepsis. Therefore, managing patients’ postoperative immune status is based on findings reported from patients with trauma, severe infection and sepsis.

The pathophysiologies of sepsis and ARDS are considered organ injuries associated with inflammatory cell infiltration mediated by systemic inflammatory responses and the subsequent release of tissue damaging mediators from inflammatory cells [3].

Systemic inflammatory response syndrome (SIRS) criteria allows the detection of early symptoms of sepsis. Patients who fulfill at least two of the following criteria are determined as SIRS: fever > 38.0 °C or < 36.0 °C, heart rate > 90 beats/min, respiratory rate > 20 breaths/min, white blood cell count > 12 × 10^9^/L or < 4 × 10^9^/L.

SIRS scores are also used to evaluate postoperative inflammatory state. SIRS score on the second postoperative day was reported to be associated with APACHE III score at the time of evaluation, length of intensive care unit stay, multiple organ failure, and mortality [4]. Therefore, it is reasonable for researchers to consider strategies to reduce SIRS scores for better outcomes.

Inhibiting inflammatory pathways including LPS binding protein, inhibiting NF-κB signaling, inhibiting adhesion molecules, and inhibiting leukocyte elastase have been investigated as therapeutic approaches for sepsis and ARDS. Although each treatment showed a promising effect in animal models, all failed to improve patient outcomes in clinical settings [5–7]. High dose steroid administration was associated with a worse outcome in a study using a large administrative database [8]. Similarly, Sivelestat sodium hydrate, a leukocyte elastase inhibitor, failed to improve the outcome for patients with pneumonia [9].

Animal studies and clinical trials reported that the mortality of sepsis increased by blocking of TNFα signaling [6]. Therefore, immune suppressive therapy might be a potential hazard that induces an immune compromised state and increases the risk of infection.

Based on accumulating observations, our understanding of the pathophysiology of sepsis has evolved from simple hyper-immunity to the time-course transition of immune status— i.e., hyper- to hypo-immunity, and therefore, it is acknowledged that the simple suppression/inhibition of hyper-immunity does not improve sepsis outcomes [10]. Previously, it was thought that compensatory anti-inflammatory response syndrome (CARS) comes after SIRS; however, this has changed to another scenario where the simultaneous expression of pro- and anti-inflammatory mediators occurs at the time of insult and the patient immune status is determined by the balance of these mediators [11]. Therefore, the immune status of each patient with sepsis varies widely based on their individual characteristics.

Similar considerations should apply for patients undergoing surgery, who were reported to enter an immune suppressive state in the early phase of postoperative periods [12], which was considered similar to that of patients with sepsis.

Inflammatory mediators induced by surgical insults are termed “damage associated molecular patterns” (DAMPs), whereas those induced by infection are called “pathogen associated molecular patterns” (PAMPs). Both signals affect innate immunity via the TLR4 signaling pathway [12].

It was reported that high-mobility group box-1, a DAMP, increases after stimulation with lipopolysaccharide, a major component of PAMPs [13]. Furthermore, bacterial translocation occurs 15% of elective surgery and associates with mortality with sepsis [14]. Therefore, it is obvious that both signals are mutually influencing each other.

Previous studies have identified preoperative/intraoperative factors that contribute to postoperative infectious complications. Patient/surgical factors associated with surgical site infection (SSI) after cesarean delivery (i.e., higher body mass index, fewer years of education, higher prior birth rate, tobacco use, prior diagnosis of hypertension, gestational diabetes, and emergency cesarean delivery) [15] and SSI in patients with Crohn’s disease receiving definitive bowel resection (i.e., lower preoperative pre-albumin, longer duration of operation and higher intraoperative lactate level) were reported [16]. However, few studies have demonstrated immune functions that link risk factors to an increase in SSI.

Our group recently reported the functional decline of alveolar macrophages after peritoneal infection in a murine model of sepsis. The host defense capacity of alveolar macrophages was impaired after peritoneal sepsis and this was associated with increased mortality after nosocomial infection. Restoring macrophage function with cytokines such as interferon (IFN)-β improved survival [17]. Furthermore, the functional decline of peritoneal macrophages occurred within 24 h after the onset of peritonitis and was restored by the systemic administration of IFN-β [18]. Therefore, some septic patients may benefit from early immune-enhancing therapy.

For patients undergoing surgery, given that the high surgical insult may lead to the situation similar to infection-induced sepsis, it is thought that early immune-enhancement may also be effective. However, our mouse model showed that the prophylactic administration of IFN-β had a worse outcome for peritoneal sepsis [18].

Therefore, the administration of immune enhancing mediators at the wrong timepoint might worsen outcomes and therefore it is crucial to identify appropriate biomarkers that can accurately distinguish patients who are about to develop immune suppression.

Expression levels of procalcitonin and HLA-DR were reported to be potential biomarkers to predict postoperative infectious complications [19]. However, in that report, the authors did not evaluate white blood cell functions and it is not clear whether they could be a biomarker to indicate the appropriate timing of immune enhancing therapy.

Therefore, it is very important to determine a patient immune status before starting therapy and more data is required to clarify the pathophysiology of immune suppression.

As perioperative physicians, we may be able to observe changes in immune status over time in more detail in patients undergoing surgery than critically ill patients. Therefore, we are in a good position to explore answers to improve the outcome of patients with immune suppression caused by highly invasive surgery, and these answers will be converted to critical care to save patients with severe infection and sepsis.
